# Anemia among pregnant women in Southeast Ethiopia: prevalence, severity and associated risk factors

**DOI:** 10.1186/1756-0500-7-771

**Published:** 2014-11-03

**Authors:** Filagot Kefiyalew, Endalew Zemene, Yaregal Asres, Lealem Gedefaw

**Affiliations:** Department of Clinical Laboratory, Bisidimo Hospital, East Harerege zone, Hararege, Ethiopia; Department of Medical Laboratory Science and Pathology, College of Public Health and Medical Sciences, Jimma University, Jimma, Ethiopia

**Keywords:** Anemia, Associated factors, Pregnant women, Southeast Ethiopia

## Abstract

**Background:**

Anemia is a significant public health problem in developing countries, particularly in pregnant women. It may complicate pregnancy, sometimes resulting in tragic outcomes. There is a lack of information on the magnitude of anemia among pregnant women in Southeast Ethiopia. The aim of this study is, therefore, to determine the prevalence of anemia and assess associated factors among pregnant women attending antenatal care (ANC) at Bisidimo Hospital in Southeast Ethiopia.

**Methods:**

A facility-based cross-sectional study, involving 258 pregnant women, was conducted from March to June 2013. Socio-demographic, medical and obstetric data of the study participants were collected using structured questionnaire. Hemoglobin was measured using a hematology analyzer and faecal specimens were examined to detect intestinal parasites. Anemia in pregnancy was defined as hemoglobin <11 g/dl.

**Results:**

Overall, prevalence of anemia was 27.9%, of which 55% had mild anemia. Rural residence (AOR =3.3, 95% CI: 1.5-7.4), intestinal parasitic infection (IPI) (AOR = 2.5, 95% CI: 1.3-4.8) and history of heavy cycle (AOR =2.7, 95% CI: 1.3-1.7) were predictors of anemia.

**Conclusions:**

This study showed moderate prevalence of anemia among the pregnant women, with a sizable proportion having severe anemia. Routine testing of pregnant women for IPIs and creating awareness on factors predisposing to anemia is recommended.

## Background

Anemia is defined as a condition in which there is less than the normal hemoglobin (Hb) level in the body, which decreases oxygen-carrying capacity of red blood cells to tissues. Anemia is a global public health problem affecting both developed and developing countries with major consequences for human health as well as social and economic development. It occurs at all stages of the life cycle [1, 2].

Anemia in pregnancy remains one of the most intractable public health problems in developing countries. Globally, anemia contributes to 20% of all maternal deaths. Although not always shown to have a causal link, severe anemia contributes to maternal morbidity and mortality [3–8]. Anemia in pregnancy may also lead to premature births [9], low birth weight [10], fetal impairment and infant deaths [11].

Apart from maternity-related complications, anemia has major consequences on human health and social and economic development. It adversely affects physical and cognitive development in children [12] and is associated with increased frailty risk in community-dwelling older adults [13]. Recently, increased risk of psychiatric disorders among children and adolescents with iron deficiency anemia has also been documented [14].

Knowledge of the relative importance of the different etiological factors forms the basis for intervention strategies to control anemia. In Sub-Saharan Africa, the causes of anemia during pregnancy are multifactorial. These include an iron and folate deficient diet and infections such as malaria, hookworms, and increasingly human immunodeficiency virus. Most of these conditions can be prevented by creating awareness and providing affordable interventions [15–18].

Anemia is a significant public health problem in Ethiopia. According to the 2012 Ethiopian Central Statistical Agency report, nationally, 44% of children aged 6-59 months were anemic, with 21%, 20% and 3% having mild, moderate and severe anemia. Moreover, 17% of women aged 15-49 were anemic; of which 13% had mild anemia, 3% were moderately anemic, and less than 1% were severely anemic. Iron deficiency anemia was ranked as one of the significant micronutrient deficiency problems in Ethiopia [19, 20].

Epidemiological studies done on prevalence of anemia in pregnant women in Ethiopia have reported varying magnitude of anemia and identified several factors associated with anemia [21–23]. Determination of the magnitude of anemia among pregnant women helps to monitor health of the pregnant women, contributing to reduction in maternal morbidity and mortality. Also, assessment of factors predisposing to anemia in a local area enables to take targeted intervention activities. Therefore, this study is aimed at determining prevalence of anemia and assessing associated risk factors among pregnant women attending antenatal care (ANC) at Bisidimo Hospital in Southeast Ethiopia.

## Methods

### Study setting

The study was conducted among pregnant women attending ANC in Bisidimo Hospital. Bisidimo Hospital is a district hospital found in East Harerege zone in Southeast Ethiopia. The hospital is located in Babile Woreda (equivalent to district), 535 Km Southeast of the capital Addis Ababa. The geographical coordinates of the district are approximately 9° 8′ 41N latitude and 42° 12′ 48E longitude with an altitude of 1357 meters above sea level. The hospital serves more than 250,000 inhabitants of the district and neighboring districts. The study was carried out from March to June, 2013.

### Study design and sampling method

A cross sectional study was conducted among pregnant women attending ANC of the hospital. A total of 258 pregnant women were enrolled in this study. Sample size was estimated using the general formula for single population proportion, with the following assumptions: anemia prevalence (P) of 21.3% [23] and using the 95% confidence level and 5% marginal error. This gave us 258; hence, all pregnant women visiting ANC during March to June 2013, were included consecutively. Pregnant women of all trimesters, who were willing to take part in the study, were included. On the other hand, pregnant women receiving therapy for anemia, severely ill thus unable to respond to the questionnaire and not willing to take part in the study were excluded.

### Data collection

Data on socio demographic, obstetric and medical history of pregnant women were collected using structured questionnaire. The questionnaire, first prepared in English and translated into the local language *Afan Oromo*, was administered by two trained nurses. Weight and height of the pregnant women were also measured for computing body mass index (BMI). Moreover, approximately 4ml of venous blood was collected using vacutainer tubes. Hematological analyses were done using CELL DYN 1800 (*Abott Laboratories Diagnostics Division*, USA). Hematological parameters measured include: Hb, mean cell volume (MCV), mean cell hemoglobin (MCH) and mean cell hemoglobin concentration (MCHC).

In this study, anemia in pregnancy was defined as Hb <11 g/dl. Also mild, moderate and severe anemia was defined as Hb measurements between 10-10.9 g/dl, 7-9.9 g/dl and less than 7 g/dl, respectively [24]. Apart from the hematological analyses, thick and thin blood films were prepared, stained using 10% Giemsa stain for 10 minutes and examined microscopically to investigate hemoparasites particularly malaria.

Also, fresh faecal specimens were collected from each study participants using a clean, leak-proof stool cups and examined for intestinal parasitic infections (IPIs) using saline wet smear and formol-ether concentration techniques, following standard procedures [25]. Two experienced microscopists examined the blood films and faecal specimens.

To assure the quality of the data, training was given to the data collectors to minimize technical and observer bias. Standard operating procedures were followed during specimen collection and laboratory procedures. Control reagents were run to check the accuracy and precision of the data generated by the hematology analyzer.

### Data analysis

Data were entered in to EPI data version 3.1 and cleaned. Finally, data were analyzed using SPSS version 20 for windows (SPSS, Chicago, IL, USA). Data were summarized in tables and figure. Bivariate and multivariate logistic regression analyses were done to identify independent predictors of anemia. Variables with p-value ≤0.25 by the bivariate analysis and other biologically plausible variables were candidates for the multiple logistic regression model. P-value was set at <0.05 for statistical significance.

### Ethical clearance

Ethical clearance was obtained from Jimma University Ethical Review Committee. Permission was obtained from Bisidimo Hospital Administration before data collection. Written informed consent was obtained from each pregnant woman prior to enrollment in the study. Individual-level obstetric and medical information obtained from the study participants was kept strictly confidential and they were assured that only aggregate data will be reported. Anemic pregnant women and pregnant women with IPIs and malaria were immediately communicated to the attending health professionals at the ANC clinic of the hospital, for treatment and follow up.

## Results

### Socio demographic characteristics of pregnant women

A total of 258 pregnant women, age ranging 18 to 37 year (mean 26.9 ± 4.8), were included in this study. Nearly half (49.2%) of the pregnant women were within the age range of 18-26 years. Many of them were married, 223 (86.4%). Majority of the pregnant women were housewives (62.4%), rural residents (60.5%) and illiterate (61.2%), who were not able to read and write at least with one language. Socio-demographic profile of the study participants is demonstrated in Table 1.

**Table 1:** **Socio-demographic characteristics and anemia among the pregnant women, Bisidimo Hospital, 2013**

Variables	Anemia diagnosis			COR (95% CI)	P-value
	Anemic	Not anemic	Total		
	No (%)	No (%)	No (%)		
**Age in years**					
18-26	35 (27.6)	92 (72.4)	127 (49.2)	1	
26-34	35 (30.7)	79 (69.3)	114 (44.2)	1.2 (0.7-2.0)	0.592
≥34	2 (11.8)	15 (88.2)	17 (6.6)	0.4 (0.1-1.6)	0.178
**Occupation**					
Housewives	41 (25.5)	120 (74.5)	161(62.4)	1.4 (0.3-6.7)	0.700
Employed	19 (32.8)	39 (67.2)	58 (22.5)	1.9 (0.4-10.0)	0.426
Farmers	10 (34.5)	19 (65.5)	29 (11.2)	2.1 (0.4-11.9)	0.399
Others*	2 (20.0)	8 (80.0)	10 (3.9)	1	
**Residence**					
Urban	17 (16.7)	85 (73.3)	102 (39.5)		
Rural	55 (35.3)	101 (64.7)	156 (60.5)	2.7 (1.5-5.0)*	0.001
**Educational status**					
Illiterate	40 (25.3)	118 (74.3)	158 (61.2)	0.7 (0.4-1.3)	0.244
Literate	32 (32)	68 (68)	100 (38.8)	1	
**Marital status**					
Married	56 (25.1)	167 (74.9)	223 (86.4)	1	
Unmarried	16 (45.7)	19 (54.3)	35 (13.6)	2.5 (1.2-5.2)*	0.014
**Body mass index**					
Low	11 (36.7)	19 (63.3)	30 (11.6)	1.9 (0.8-4.4)	0.135
Normal	30 (31.6)	65 (68.4)	95 (36.8)	1.5(.8-2.7)	0.166
High	31 (23.3)	102 (76.7)	133 (51.6)	1	

*Statistically significant at P < 0.05; CI: Confidence interval; COR: crude odds ratio, others*: Daily laborers and Merchants.

### Obstetric and medical history of pregnant women

**Figure 1:**
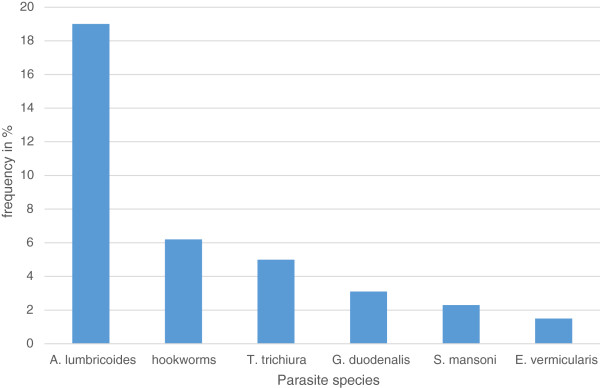
**Prevalence of intestinal parasites identified among the pregnant women, Bisidimo hospital, Southeast Ethiopia, 2013.**

Over half of the pregnant women, 134 (51.9%) were in their second trimester of pregnancy. Thirty seven (14.3%) of the pregnant women responded to have previous miscarriage. Majority of the pregnant women (80.2%) were multigravidae. More than a third of the pregnant women (37.2%) were infected with intestinal parasites (Table 2). A total of six species of intestinal parasite were identified. *Ascaris lumbricoides* was the predominant (19%) intestinal parasite identified followed by the hookworms (6.2%). Prevalence of each intestinal parasite is displayed in Figure 1. Moreover, *Plasmodium* species were detected in 9 (3.5%) of the total blood films examined, 7 of the cases were due to *Plasmodium vivax* and the remaining two cases were due to *Plasmodium falciparum*.

**Table 2:** **Clinical variables in association with anemia among the pregnant women, Bisidimo Hospital, 2013**

Variables	Anemia diagnosis			COR (95% CI)	p-value
	Anemic	Not anemic	Total		
	No (%)	No (%)	No (%)		
**History of miscarriage**					
Yes	9 (24.3)	28 (75.7)	37 (14.3)	0.8 (0.4-1.8)	0.600
No	63 (28.5)	158 (71.5)	221 (85.7)	1	
**History of heavy cycle**					
≤5 days	45 (25.0)	135 (75.0)	180 (69.8)	1	
>5days	27 (34.6)	51 (65.4)	78 (30.2)	1.6 (0.9-2.8)	0.115
**Trimester**					
First	14 (30.4)	32 (69.6)	46 (17.8)	1	
Second	29(21.6)	105 (78.4)	134 (51.9)	0.6 (0.3-1.3)	0.230
Third	29 (37.2)	49 (62.8)	78 (30.2)	1.4 (0.6-2.9)	0.447
**Parity**					
0	10 (19.6)	41 (80.4)	51 (19.8)	1	
1-4	55 (30.4)	126 (69.6)	181 (70.2)	1.8 (0.8-3.8)	0.134
>5	7 (26.9)	19 (73.1)	26 (10.1)	1.5 (0.5-4.6)	0.466
**Birth interval (n = 211)**					
≤2 years	56 (30.1)	(69.9)	186 (88.2)	1.4 (0.5-3.6)	0.530
>2 years	6 (24.0)	19 (76.0)	25 (11.8)	1	
**Red meat/poultry/fish consumption**					
Yes	22 (31.4)	48 (68.6)	70 (27.1)	1	
No	50 (26.6)	138 (73.4)	188 (72.9)	1.3 (0.7-2.3)	0.442
**Fruit/vegetable consumption**					
Yes	22 (26.2)	62 (73.8)	84 (32.6)	1	
No	50 (28.7)	124 (71.3)	174 (67.4)	0.9 (0.5-1.6)	0.880
**Intestinal parasite detected**					
Yes	40 (41.7)	56 (58.3)	96 (37.2)	1.9 (1.1-3.4)*	0.019
No	40 (24.7)	122 (75.3)	162 (62.8)	1	
**Malaria infection**					
Yes	3 (33.3)	6 (66.7)	9 (3.5)	0.3 (0.4-2.6)	0.278
No	77 (30.9)	172 (69.1)	249 (96.5)	1	

*Statistically significant at P < 0.05.

### Prevalence of anemia among pregnant women

The overall prevalence of anemia in this study was 27.9%. The mean Hb value was 11.4 ± 2.3 g/dl. Of the anemic pregnant women, 55%, 32.5% and 12.5% had mild, moderate and severe anemia, respectively.

The prevalence of anemia was higher (34.6%) in pregnant women in the age group of 18-26 years; however, the difference was not significant. Majority of the study participants were rural residents with significantly (P = 0.001) higher prevalence of anemia compared to their urban counterparts. Anemia in those infected with intestinal parasites was significantly higher (P = 0.019) than the non-infected ones. Similarly, prevalence of anemia was significantly higher (AOR = 2.7, 95% CI: 1.3-1.7) in pregnant women who reported history of heavy cycle (Table 2).

### Associated factors of anemia

After adjusting for other variables: residence in rural area (AOR = 3.3, 95% CI: 1.5-7.4), intestinal parasitic infection (IPI) (AOR = 2.5, 95% CI: 1.3-4.8) and history of heavy menstrual cycle (>5days of menses) (AOR = 2.7, 95% CI: 1.3-1.7) were the predictors of anemia among the pregnant women (Table 3).

**Table 3:** **Predictors of anemia among the pregnant women, Bisidimo Hospital, 2013**

Variable	COR (95% CI)	P-value	AOR (95% CI)	P-value
**Age in years**				
18-26	1	1		
26-34	1.2 (0.7-2.0)	0.592	1.7 (0.9-3.2)	0.127
≥34	0.4 (0.1-1.6)	0.178	0.4 (0.1-1.8)	0.202
**Residence**				
Urban	1	1		
Rural	2.7 (1.5-5.0)	0.001*	3.3 (1.5-7.4)*	0.003
**Marital status**				
Married	1	1		
Unmarried	2.5 (1.2-5.2)	0.014	2.0(0.8-4.6)	0.113
**Educational status**				
Illiterate	0.7 (0.4-1.3)	0.244	0.6 (0.3-1.2)	0.153
Literate	1	1		
**History of heavy cycle**				
≤5 days	1	1		
>5days	1.6 (0.9-2.8)	0.115	2.7 (1.3-1.7)*	0.006
**Trimester**				
First	1			
Second	0.6 (0.3-1.3)	0.230	1.3 (0.5-3.2)	0.639
Third	1.4 (0.6-2.9)	0.447	1.6 (0.7-4.1)	0.289
**Parity**				
0	1		1	
1-4	1.8 (0.8-3.8)	0.134	1.3 (0.5-3.2)	0.639
>5	1.5 (0.5-4.6)	0.466	1.6 (0.7-4.1)	0.289
**Intestinal parasite detected**				
Yes	1.9 (1.1-3.4)	0.019	2.5 (1.3-4.8)*	0.007
No	1		1	
**Body mass index**				
Low	1.9 (0.8-4.4)	0.135	2.4 (0.9-6.3)	0.084
Normal	1.5 (.8-2.7)	0.166	1.7 (0.9-3.2)	0.134
High	1		1	

*Statistically significant at <0.05 AOR: Adjusted Odds Ratio, adjusted for other variables in the table CI: Confidence interval COR: Crude Odds Ratio.

## Discussion

The overall prevalence of anemia among pregnant women was 27.9%. According to WHO classification of the public health importance of anemia [24], it is a moderate public health problem among the pregnant women in our study. Out of the anemic pregnant women, however, 12.5% had severe anemia, Hb concentration of below 7 mg/dl.

The overall prevalence of anemia obtained in this study is higher than reports from Gondar (21.6%) [26], Nigeria (23.2%) [18] and Turkey (27.1%) [27]. This might be due difference in the socio-demographic factors and lack of awareness about the consequences of anemia in our study participants. In this study, pregnant women on iron supplementation were not included, while these were included in studies done in Gondar and Turkey. In the study done in Nigeria [18], it has been indicated that use of hematinics and antimalarial drugs is a common practice in Nigeria. This may possibly lower the prevalence of anemia among the pregnant women in Nigeria compared to the pregnant women in our study.

Higher magnitudes of anemia were reported from Arsi (36.6%) [28], Addis Ababa (33%) [23], and around Gilgel Gibe dam area in southwest Ethiopia (53.9 %) [29], Eastern Sudan (62.6%) [30]. This might be due to variation in sample size and presence of high malaria infection. For instance the prevalence of malaria in the study done around Gilgel Gibe area (11.6%) and eastern Sudan (reported 13.7% of *P. falciparum* malaria) were relatively high which might have contributed to the high prevalence of anemia.

In this study, mild anemia was common followed by moderate anemia. Similar findings were reported in other local studies [23, 29] and the study done in Nigeria [18].

Heavy infection with soil-transmitted helminthes (STHs), particularly the hookworms, predisposes pregnant women and individuals with low iron store to anemia. In this study, more than a third of the pregnant women were infected with intestinal parasites, *Ascaris lumbricoides* and the H*ook worms* being predominant. Pregnant women with IPIs were 2.5 times more likely to be anemic compared to their non-infected counterparts. Other local studies [26, 29] also documented similar findings.

A significantly higher prevalence of anemia was found among pregnant women who were from rural areas. Pregnant women from rural areas were more than three times more likely to be anemic than their urban counterparts. Association of rural residence with anemia has also been reported earlier [26]. The higher prevalence of anemia among pregnant women from rural areas is likely related to lack of information about adequate nutrition during pregnancy, economic factors and inaccessibility of health care centers.

Pregnant women with a history of heavy cycle were more anemic than those with normal menstruation cycle. In this study pregnant women with a history of heavy cycle were 2.7 times more likely to be anemic than those who had normal menstruation cycle (AOR = 2.7, 95% CI: 1.3-1.7).

In this study, the association of other obstetric and dietary habits with anemia was also assessed (Table 2). Accordingly, no significant association of history of miscarriage, parity, trimester, birth interval, body mass index, dietary habit, educational status and occupation with anemia was obtained. In contrast, association of anemia with multiparity and intake of vegetables and fruits was reported in the study done in Arsi [28]. This is in contrary with studies done by Alem *et al.* [26], and Karaoglu *et al.* [27]. This might be due to variation in method and study subject involved.

Malaria infection during pregnancy is life-threatening. In this study, nine of the pregnant women were malaria positive by thick film microscopy. It is likely that this magnitude of malaria is underestimated in this study, as submicroscopic *Plasmodium* infections are missed by the diagnostic method used [31]. Most of the detected cases (seven out of nine) were due to *Plasmodium vivax*. Malaria infection during pregnancy in the area calls urgent intervention activities targeting pregnant women to be in place to prevent tragic outcomes of the infection during pregnancy [32, 33]. In Ethiopia, almost all cases of malaria are caused by *P. falciparum* and *P. vivax*, the former causing about 60% of malaria cases. Major transmission of malaria occurs in Ethiopia from September to December, following the major rains. Minor transmission occurs from April to May following the minor rains. In Ethiopia, *P. vivax* is relatively common during the minor transmission periods, which is also observed in this study.

The findings of this study should be interpreted with caution due to the following limitations of the study. First, due to the cross sectional study design used, whether anemia preceded the predisposing factors or the vice versa could not be verified in this study. We exclude those severely ill pregnant women and unable to respond due to difficulty of getting venous sample. This may potentially reduce the prevalence of anemia. Third, micronutrients were not measured in this study, which limits further classification of the anemia. Last, the worm burden of the STHs had not been determined. As a result, severity of the helminths infections could not be estimated.

## Conclusion

The prevalence of anemia among pregnant women is moderate, however, sizable proportion of the pregnant women had severe anemia. Pregnant women residing in rural areas, having IPIs and history of heavy cycle had a higher risk of anemia. Awareness creation on the consequences of anemia during pregnancy should be given to women in child bearing age in general and pregnant women in particular. Routine screening and deworming of pregnant women infected with intestinal parasites is recommended.
